# A New Mutation in *IDS* Gene Causing Hunter Syndrome: A Case Report

**DOI:** 10.3389/fgene.2019.01383

**Published:** 2020-03-18

**Authors:** Caio Perez Gomes, Maryana Mara Marins, Fabiana Louise Motta, Sandra Obikawa Kyosen, Marco Antonio Curiati, Vânia D’Almeida, Ana Maria Martins, João Bosco Pesquero

**Affiliations:** ^1^Center for Research and Molecular Diagnosis of Genetic Diseases, Department of Biophysics, Escola Paulista de Medicina, Universidade Federal de São Paulo, São Paulo, Brazil; ^2^Inborn Errors of Metabolism Reference Center, Department of Pediatrics, Escola Paulista de Medicina, Universidade Federal de São Paulo, São Paulo, Brazil; ^3^Inborn Errors of Metabolism Laboratory, Department of Psychobiology, Escola Paulista de Medicina, Universidade Federal de São Paulo, São Paulo, Brazil

**Keywords:** mucopolysaccharidosis type II, *IDS*, iduronate-2-sulfatase, Hunter syndrome, inborn errors of metabolism, lisossomal storage disease

## Abstract

**Rationale:**

Mucopolysaccharidosis type II (Hunter syndrome) is an X-linked multisystem disorder, caused by deficiency of the lysosomal enzyme iduronate-2-sulfatase (I2S). The clinical manifestations of this disease are severe skeletal deformities, airway obstruction, cardiomyopathy, and neurologic deterioration.

**Patient:**

The patient was 5 years and 6 months boy, with developmental delay, hearing loss, hepatosplenomegaly, and skeletal dysplasia. He was diagnosed with mucopolysaccharidosis type II based on clinical manifestations, biochemical and genetic analysis.

**Outcomes:**

The patient carries a new mutation (c.879-1210_1007-218del) in hemizygosis in the *IDS* gene, which was defined as pathogenic according to the 2015 American College of Medical Genetics and Genomics-Association for Molecular Pathology guidelines and as responsible for the mucopolysaccharidosis type II phenotype in the patient.

## Introduction

Hunter syndrome, also known as mucopolysaccharidosis type II (MPS II), is a rare X-linked recessive disorder caused by deficiency of the lysosomal enzyme iduronate-2-sulfatase (*IDS* gene—OMIM 309900), leading to progressive accumulation of glycosaminoglycans (Neufeld and Muenzer, 1995; Wraith et al., 2008; Roberts et al., 2016; Tomatsu et al., 2018).

Children with Hunter syndrome present a variety of clinical symptoms, ranging from attenuated to severe. The distinguishing factor of severity is the presence, or absence, of progressive cognitive deterioration; the severe form affects approximately 75% of all MPS II patients (Neufeld and Muenzer, 1995; Wraith et al., 2008; Jones et al., 2009; Quaio et al., 2012; Roberts et al., 2016).

Patients with mild phenotype generally have normal cognitive function and consequently extended survival compared to those with the severe form, with progressive cognitive deterioration (Neufeld and Muenzer, 1995; Wraith et al., 2008; Jones et al., 2009; Roberts et al., 2016).

The majority of clinical symptoms are severe airway obstruction, skeletal deformities, hepatosplenomegaly, cardiomyopathy, and in most patients, neurologic decline (Neufeld and Muenzer,1995). The treatment for the disease usually consists of enzyme replacement therapy and palliative care to manage disease symptoms and the effects of cognitive and somatic deterioration (Jones et al., 2009; Roberts et al., 2016).

The *IDS* gene is located on the X*q*28 chromosome and 640 mutations related to Hunter Syndrome are reported on the Human Gene Mutation Database (HGMD). From the mutations described, 323 are missense or nonsense, 59 splicing substitutions, 119 small deletions, 49 small insertions/duplications, 14 small indels, 52 gross deletions, 4 gross insertions/duplications, and 20 complex rearrangements (Stenson et al., 2003).

The aim of our study is to describe a new mutation in the *IDS* gene causing Hunter syndrome.

## Case Presentation

**Figure 1:**
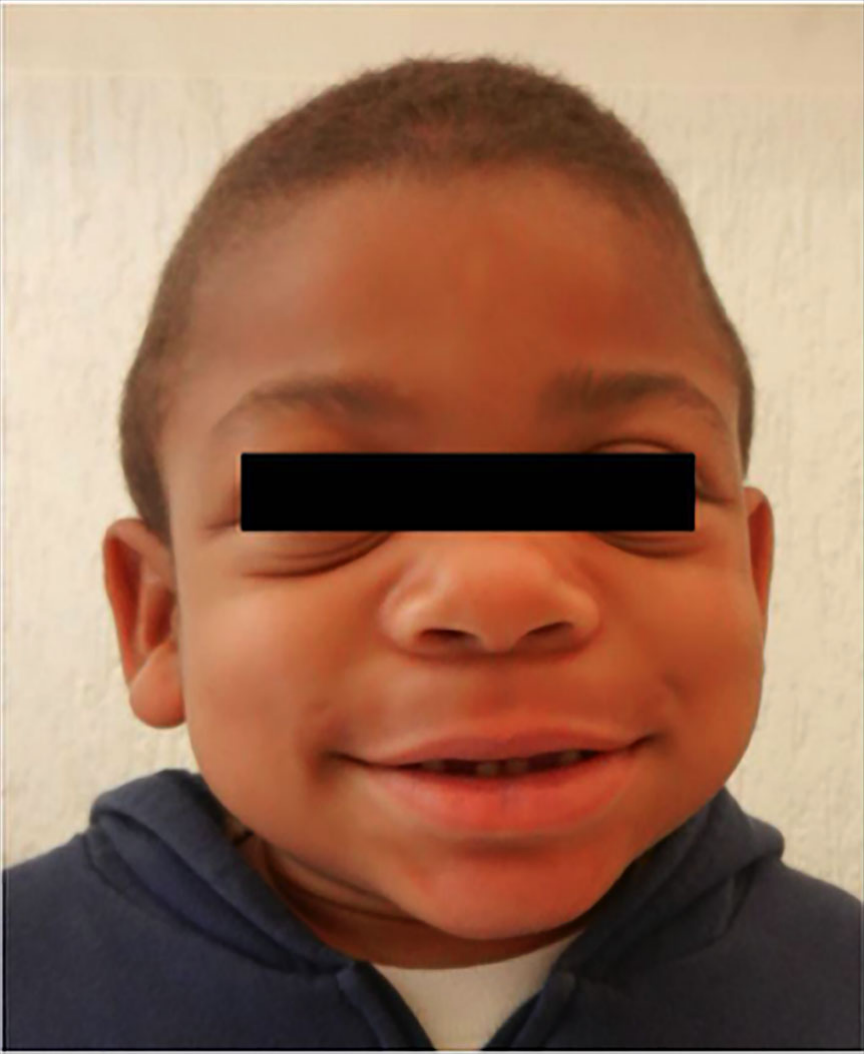
Patient's dysmorphic facial features—broad nasal bridge, large rounded cheeks, and thick large lips.

Patient was referred at the age of 5 years and 6 months for investigation by the pediatrician due to hepatosplenomegaly, developmental delay, and coarse facial features (**Figure 1**). He was born from unrelated parents, has one older healthy sister and negative familial history for MPS II. Mother had uneventful pregnancy, cub-foot noted at birth, no intercurrent illness during neonatal period, but parents refer that he always had coarse facial features. At the age of 1 year and 6 months he was hospitalized due to severe wheezing and he had other three hospitalizations for the same reason. At the same age he had the onset of snoring, sleep apnea, and recurrent upper airway tract infections.

At the age of 2 years the patient presented umbilical and bilateral inguinal hernias; after 6 months developed hepatosplenomegaly and when he was 3 years old presented stiffness of hands. The patient had developmental delay; he walked unassisted at 1 year and 10 months of age and started talking at 3 years old. At his first appointment at our service, it was evidenced cognitive impairment and the classic phenotypic signs of MPS II as coarsening of the facial features, hepatosplenomegaly, umbilical hernia, bilateral inguinal hernia, claw hands, and stiff articulations.

**Figure 2:**
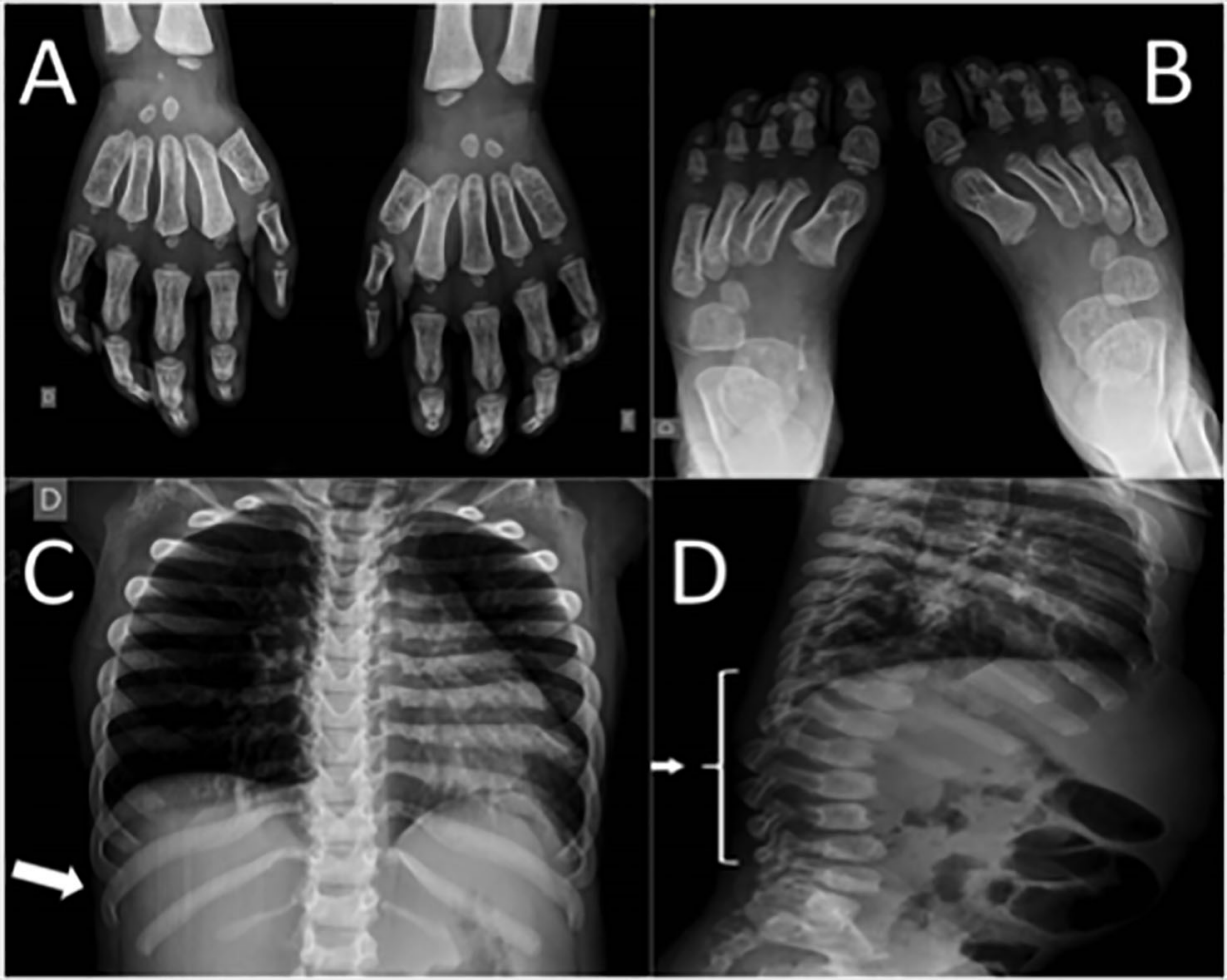
Patient's radiographic findings. **(A)** Proximal pointed phalanges of hands. **(B)** Proximal pointed phalanges of feet. **(C)** Widened anterior and tapered posterior ribs (arrow). **(D)** “Beaked vertebra” of lumbar spine (arrow).

Neurologic examination showed appendix spasticity in lower limbs, impaired gait due to joint mobility, thoracic scoliosis, delayed speech, and agitation. Goniometry evaluation revealed impaired range of motion in upper and lower limbs. Radiographic findings (**Figure 2**) were consistent with dysostosis multiplex with widened anteriorly and tapered posteriorly ribs (“paddle-shaped” ribs), malformation of lumbar vertebral bodies (“beaked vertebra”), and proximal pointed phalanges of hands and feet (“bullet-shaped phalanges”). Ecochardiography revealed mitral valve thickness with mild insufficiency, mild tricuspid valve regurgitation, severe aortic insufficiency, mild left ventricular hypertrophy, mild pericardial effusion—patient was referred to cardiologist who also detected systemic hypertension and prescribed captopril and furosemide. Audiometric evaluation showed moderate conductive hearing loss. Polysomnography detected increased apnea-hypopnea index (104 events/h), oxyhemoglobin saturation of 88–91% throughout the exam (low limit of normality), oxyhemoglobin desaturation associated to obstructive events. Evaluation with otolaryngologist revealed adenoid hypertrophy (60% of enlargement) and tonsils enlargement (grade IV).

## Materials and Methods

### Subject

The approval for the use of the medical records for retrospective studies of this case study (CEP-UNIFESP #2007/11) was provided by the Ethics Committee of the Universidade Federal de São Paulo, São Paulo, Brazil.

## Methods

The first morning void urine samples were obtained for uGAG analyses each day the patient came to the institution to receive enzyme replacement therapy (ERT). Urinary GAG excretion was determined in the urine sample of the patient according to the technique described by Jong et al., (1989), which is a color reaction with dimethylmethylene blue. The upper limits for the normal uGAG levels were defined according to age as follows: < 1 year, 1–2 years, 2–4 years, 4–6 years, 6–10 years, 10–15 years, 15–20 years, and 20–50 years (Jong et al., 1989). The uGAG levels were correlated with urinary creatinine, which was measured using the Biotécnica^®^ kit (Biotécnica Indústria e Comércio Ltda, Varginha, MG, Brasil).

The I2S activity from dried blood spot was performed by fluorimetric assay with enzyme artificial substrate 4-methylumbelliferyl-α-iduronate-2-sulfate, according to Rezende et al. (2014).

Blood sample from patient for genetic analysis was collected in EDTA (DNA) and Tempus tube (RNA), the DNA was isolated from total blood using QIAamp DNA blood mini kit (Qiagen, Valencia, CA, USA). The RNA was isolated from Tempus tube using Tempus Spin RNA Isolation Kit and reverse transcripted by Superscript III reverse transcriptase kit (Thermo Fisher Inc.). The total RNA was reverse transcripted with random hexamer primers.

The DNA and complementary DNA (cDNA) were submitted to PCR using specific primers with GoTaq Green Master MIX (Promega Inc). The long range PCR was performed using GoTaq Long PCR Master MIX (Promega Inc).

The primers (Exxtend Biotech) were designed to specific regions, not binding or amplifying the pseudogene. The primers sequences are shown in the **Supplementary Material** (**Table S1**). Amplicons were analyzed by electrophoresis in 1% agarose gel and purified from gel with QIAquick gel extraction kit (QIAGEN Inc). The sequencing reaction was performed with BigDye Terminator v3.1 cycle sequencing kit (Applied Biosystems Inc), in accordance with manufacturer's protocol. Fragments were sequenced by the automatic DNA sequencer, Genetic analyzer 3500 XL (Applied Biosystems Inc), the alignment of the electropherograms produced was performed against the reference sequence of the *IDS* gene (Accession Number NG_011900 and NM_000202) with Geneious R10 software (Biomatters Inc). We analyzed the coding exons and their flanking regions.

## Results

### Biochemical and Molecular Analysis

The patient presented an altered biochemical profile, with urinary glycosaminoglycan (GAG) of 15.03 mg GAG/mmol creatinine (normal reference for the age of 4 to 6 years is <9.2 mg GAG/mmol creatinine) (Jong et al., 1989). In addition, the lysosomal enzyme I2S activity obtained from dried blood spot was 0.73 μmol/L blood/h (normal reference is > 4.42 μmol/L blood/h) (Rezende et al., 2014).

**Figure 3:**
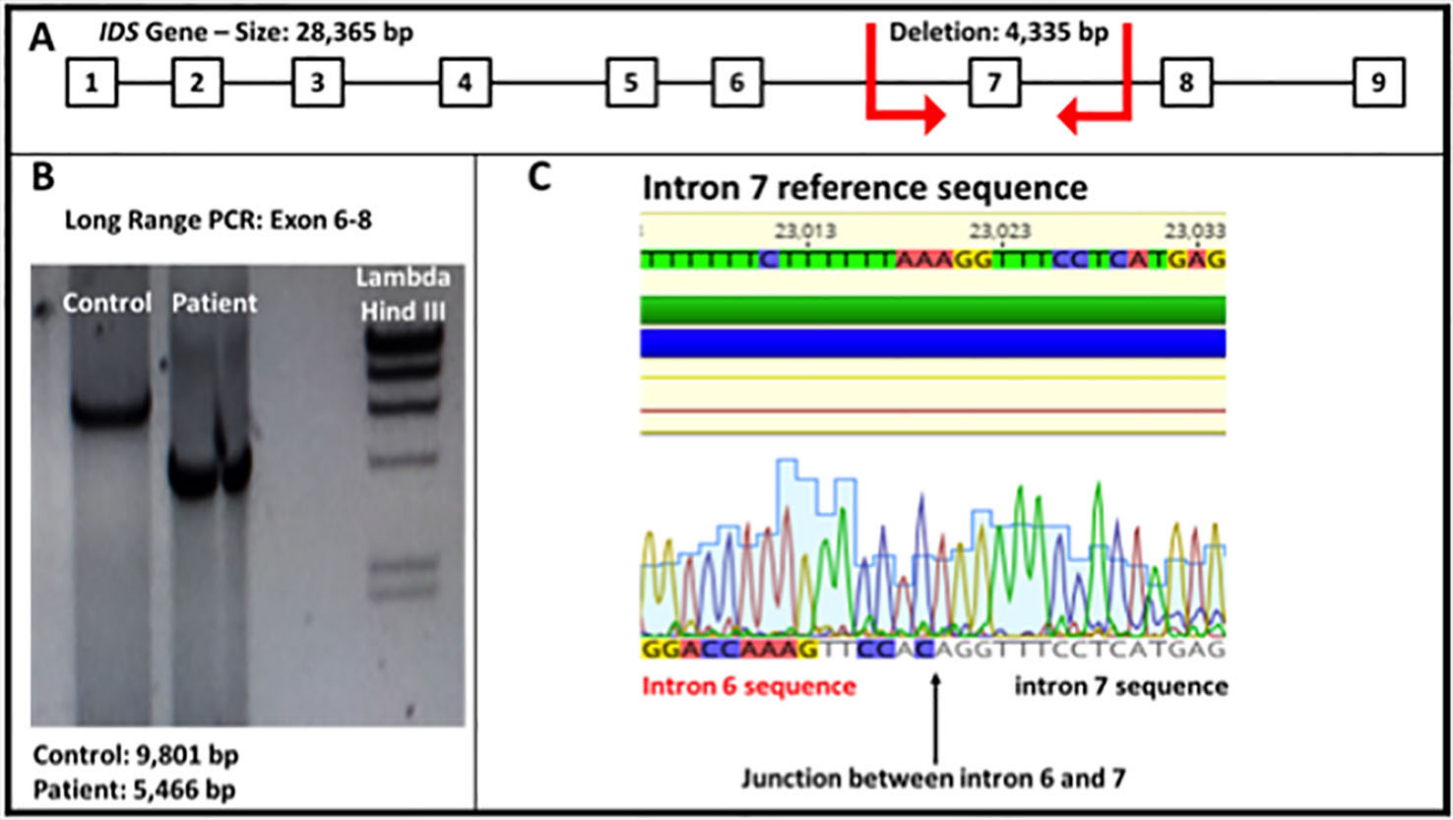
**(A)** *IDS* gene molecular structure. The red arrows show the breakpoints of the deletion observed in the patient (4,335 bp). **(B)** Electrophoresis of a long range PCR, showing the amplified fragment spanning from exon 6 to 8 (Fragment size: normal control 9,801 bp and patient 5,466 bp). **(C)** Sequence obtained from the genomic DNA of the patient shows the junction between intron 6 and 7 of the *IDS* gene (total deletion of exon 7).

For the genetic testing, the genomic DNA was isolated from total blood of the patient. Using specific oligonucleotides, the coding exons and their flanking regions for the *IDS* gene were amplified and sequenced by the Sanger sequencing methodology. Molecular analysis of the obtained sequences revealed three variants. The first variant (p.T146T, c.438C > T, rs1141608) is classified as benign (Stenson et al., 2003) and the second variant (p.T214M, c.641C > T, rs61736892) has conflicting interpretations of pathogenicity (Sherry et al., 2001; Stenson et al., 2003). The third variant was discovered due to the fact that the fragment containing exon 7 could not be amplified by PCR using specific primers. A long range PCR from exon 6 to 8 amplified a smaller fragment compared to the control fragment. Sanger sequencing revealed a new mutation (c.879-1210_1007-218del), causing a deletion ranging from intron 6 to intron 7, containing the whole exon 7 (**Figure 3**). This mutation has never been observed before, but the complete deletion of exon 7 has been described in a Colombian patient and classified as pathogenic (Stenson et al., 2003; Galvis et al., 2015).

**Figure 4:**
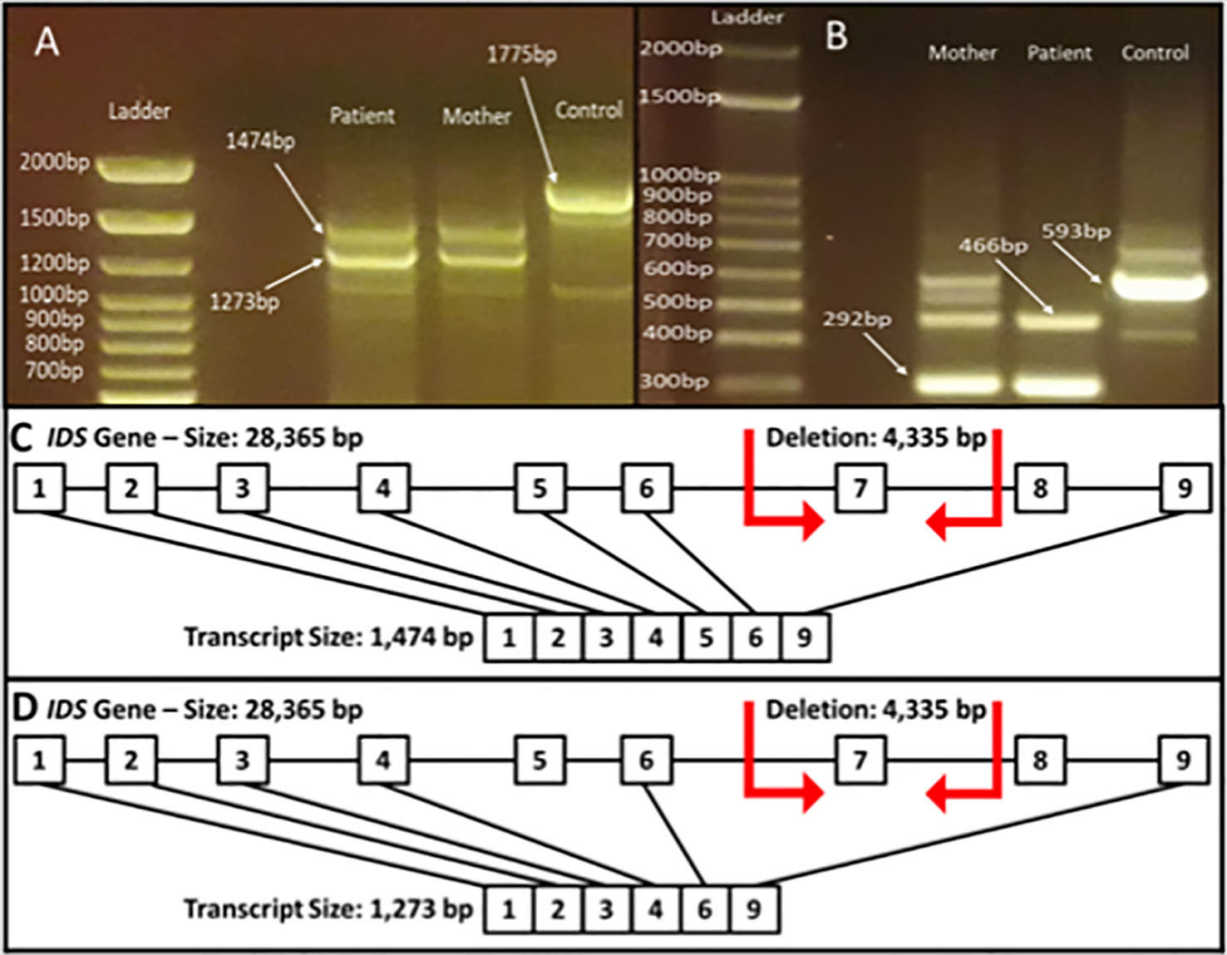
Reverse transcriptase (RT) for IDS gene from total blood RNA obtained from the patient and his mother. **(A)** PCR fragment from exons 1–9 (expected fragment size 1,775 bp). It is possible to observe the generation of the fragments lacking exons 7 and 8 (1,474 bp) and fragment lacking exons 5, 7, and 8 (1,273 bp). **(B)** PCR fragment from exons 6 to 9 (expected fragment size 593 bp). It is possible to observe the generation of the fragments lacking exon 7 (466 bp) and fragment lacking exons 7 and 8 (292 bp). The figure depicts the transcript of 1,474 bp lacking exons 7 and 8 **(C)** and the transcript of 1,273 bp lacking exons 5, 7, and 8 **(D)**.

In order to evaluate the impact of this deletion on gene expression, we investigated the RNA expression of the *IDS* gene in white blood cells from the patient and his mother. The expression of two aberrant transcripts were detected: one lacking exons 7 and 8 and another one lacking exons 5, 7, and 8 (**Figure 4**). It is possible to observe that the wild type transcript (1,775 bp) could not be observed in the mother (**Figure 4A**). This result could be explained by at least two reasons: a PCR on cDNA of different lengths is more difficult for the longest template, or the corresponding wild type mRNA of the mother is less expressed due to X-inactivation, and thus the signal is lost. Therefore we performed another PCR analysis using an internal primer mapping in the exon 6, which generates a shorter band of 593 bp also in the sample from the mother (**Figure 4B**). As the figure shows, in this condition the band corresponding to the wild type transcript could be observed also in the mother.

## Discussion and Conclusion

The mucopolysaccharidosis type II or Hunter syndrome is a rare X-linked disease with an incidence of 0.62 per 100.000 births in Australia, 0.31 western Australia, 0.64 Germany, 0.71 Northern Ireland, and 0.30 in British Columbia (Sherry et al., 2001). In Brazil, a study evaluated patients with lysosomal storage diseases over a 14-year period and estimated the frequency of MPS II patients in 0.71 among the births in this period (Giugliani et al., 2017). In Latin America, no official data on the incidence of MPS diseases is available (Martin et al., 2008). The disease is caused by the accumulation of glycosaminoglycans, due to deficiency of the enzyme iduronate-2-sulfatase (Roberts et al., 2016).

The patients exhibit a variety of clinical symptoms, from mild to severe (Jones et al., 2009; Roberts et al., 2016; Tomatsu et al., 2018). Death may occur in the second decade of life, although, patients with minimal or no neurologic involvement may reach adulthood; the milder variants may survive up to the fifth and sixth decades (Martin et al., 2008; Gupta et al., 2015).

The patients need multidisciplinary care to treat the disease symptoms and the enzyme replacement therapy help several patients. However, the recombinant enzyme does not cross the blood-brain barrier, therefore the enzymatic therapy does not act in the central nervous system; patients affected by the neurological form of the disease do not have an effective treatment (Giugliani et al., 2014).

The clinical manifestations of MPS II in our patient were similar to the vast majority of symptoms present in the severely affected patients worldwide, presenting low levels of the enzyme I2S activity in the blood, as well as high amounts of GAGs. The genetic cause for the disease was shown to be the variant c.879-1210_1007-218del in the *IDS* gene, a deletion described for the first time in the literature. The breakpoints of this deletion occur between introns 6 and 7, causing the complete deletion of exon 7. Based on DNA analysis, this deletion creates a premature stop codon at the fourth amino acid of exon 8 and therefore, the protein generated from this transcript should not have the amino acids from exon 7 due to deletion and the amino acids of exon 8 and 9 due the premature stop codon. However, based on our RNA results it is possible to observe that this genomic deletion promotes two alternative splicing of the gene, producing transcripts lacking exons 5 and 8, in addition to exon 7 (**Figure 4**). Several studies have shown complex rearrangements that lead to gross deletions in the *IDS* gene. The exon 7 deletion was previously reported in a Colombian patient (Galvis et al., 2015), however without describing the breakpoint of the mutation. Other studies have shown deletions and rearrangements involving the exon 7. A French study showed a severely affected patient carrying a gross deletion spanning the exons 4, 5, 6, and 7 of the IDS gene (Birot et al., 1996). In another study with Israeli families (Simon-Schiff et al., 1994), the authors identified two patients with deletions of the exons 5, 6, and 7. Oshima et al. in 2011, identified two cases of rearrangements that included a partial deletion of *IDS* gene and an inverted insertion of the pseudogene *IDSP1*, probably mediated by an Alu element (Oshima et al., 2011).

The analysis of the DNA of our patient revealed a common sequence (5'AGGTTTC) present in the genomic junction. This sequence is present at both ends, flanking the deleted region (**Figure 1****-****Supplementary material**), and it is present in other parts of the gene, like the 5' region, intron 3, 4, 6 (two times), intron 7 (three times), and 3'UTR. We believe this sequence could act as an Alu-like element, generating a susceptible site for an undefined genetic event (recombination and deletion), as observed in other genes (Amiñoso et al., 2013; Happ et al., 2016; Garland et al., 2017).

In summary, we can conclude that the cause for the MPS II in this patient is a gross deletion spanning from intron 6 to intron 7, leading to the total deletion of exon 7. Interestingly this deletion produces aberrant transcripts without exons 5, 7, and 8 at the transcriptional level. The clinical symptoms, biochemical and genetic findings lead to the conclusion that this mutation is pathogenic. Therefore, this is a new variant in the *IDS* gene and the knowledge of its pathogenicity is pivotal for molecular diagnosis, treatment of the patients and genetic counseling of the relatives.

## Data Availability Statement

The raw data supporting the conclusions of this manuscript will be made available by the authors, without undue reservation, to any qualified researcher.

## Ethics Statement

The studies involving human participants were reviewed and approved by Ethics Committee of the Universidade Federal de São Paulo, São Paulo, Brazil. Written informed consent to participate in this study was provided by the participants' legal guardian/next of kin. Written informed consent was obtained from the minor(s)' legal guardian/next of kin for the publication of any potentially identifiable images or data included in this article.

## Author Contributions

CG and JP designed the study and drafted the manuscript with the assistance of SK. CG, MM, and FM performed the genetic analysis and bioinformatics evaluations. SK, MC, and AM conducted the clinical evaluations. SK and VD’A performed the biochemical analysis. All authors analyzed the data and approved the final manuscript.

## Funding

This work was supported by the Coordenação de Aperfeiçoamento de Pessoal de Nível Superior - Brasil (CAPES) - Finance Code 001 (fellowships for CG and FM) and by a grant from the São Paulo State Foundation (Process 2014/27198-8 FAPESP).

## Conflict of Interest

The authors declare that the research was conducted in the absence of any commercial or financial relationships that could be construed as a potential conflict of interest.

## Abbreviations

MPS II, mucopolysaccharidosis type II; I2S, iduronate-2-sulfatase; HGMD, Human Gene Mutation Database; GAG, glycosaminoglycan.
