# Supplementary material for: A New Mutation in IDS Gene Causing Hunter Syndrome: A Case Report
Source: Front Genet. 2020 Mar 18;10:1383. doi: 10.3389/fgene.2019.01383 (PMC7093562; doi:10.3389/fgene.2019.01383)
Supplement: Supplementary file 1 [file Presentation_1.pptx]

## Slide 1
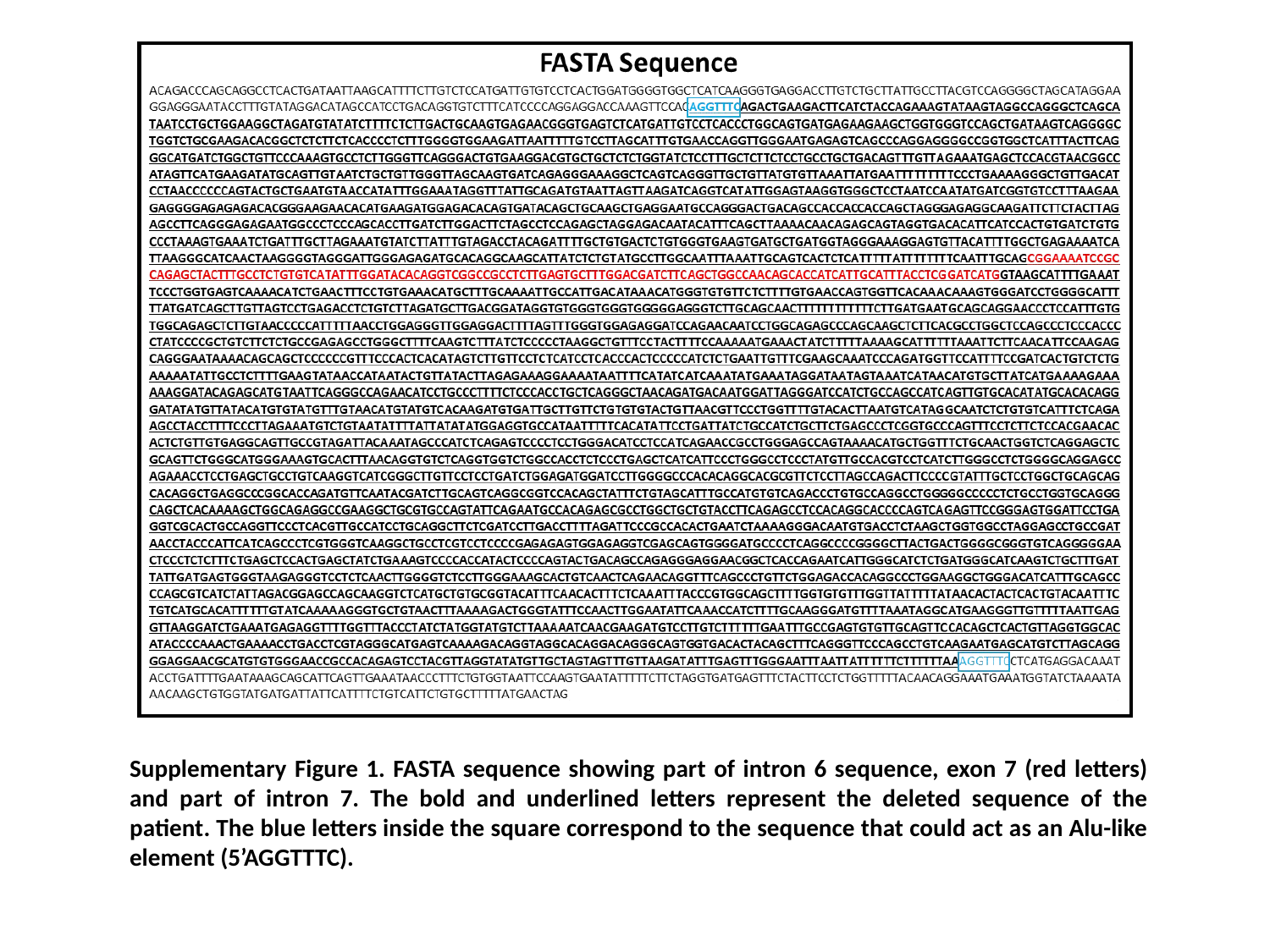

Supplementary Figure 1. FASTA sequence showing part of intron 6 sequence, exon 7 (red letters) and part of intron 7. The bold and underlined letters represent the deleted sequence of the patient. The blue letters inside the square correspond to the sequence that could act as an Alu-like element (5’AGGTTTC).
