# Supplementary material for: A New Mutation in IDS Gene Causing Hunter Syndrome: A Case Report
Source: Front Genet. 2020 Mar 18;10:1383. doi: 10.3389/fgene.2019.01383 (PMC7093562; doi:10.3389/fgene.2019.01383)
Supplement: Supplementary file 2 [file Table_1.docx]

**Supplementary material**.

**Table 1.** Primers sequences used in PCRs assays.

| **Primers for DNA fragments** | | | |
| --- | --- | --- | --- |
| **Fragment**  **Size (bp)** | **Primer Forward**  **5’ – 3’** | **Primer Reverse**  **5’ – 3’** | ***IDS* gene site** |
| **405** | (1F) TAACTGCGCCACCTGCTGC | (1R) GAAGCACGGAGGGATAGGAG | Exon 1 |
| **375** | (2F) CTGGCTAGGCTGTTAAGGTGC | (2R) CCCTCTAACAAGATGTCCCGC | Exon 2 |
| **811** | (3F) GTTTGCTAGGAGCCTCGGG | (3R) TAAGGAATGTCCGTCTCTGGG | Exon 3 |
| **373** | (4F) CTTAGGGACCAGGAAGTCAG | (4R) CACAGAACATGCAGTATACCC | Exon 4 |
| **399** | (5F) TGACTAACACGTGAAGGGCTG | (5R) TAGCCACCTTCCCTGTGCA | Exon 5 |
| **312** | (6F) AGTATAGACAGTGATAGAGCCAC | (6R) GTTTCACCTACGACACTATGTC | Exon 6 |
| **369** | (7F) ATTATCTCTGTATGCCTTGGC | (7R) GGTCTCAGGACTAACAAGCTG | Exon 7 |
| **608** | (8F) CCGCCACAGAGTCCTACGTT | (8R) GAAATTCCAACACAAGGCAGG | Exon 8 |
| **605** | (9F) TTACTGCTCCTGCCTGGC | (9R) ACTCCTCCTCTCACCAGCTGG | Exon 9 |
| **Primers for RNA fragments** | | | |
| **1775** | (1F) TAACTGCGCCACCTGCTGC | (9R) ACTCCTCCTCTCACCAGCTGG | Exons 1, 9 |
| **593** | (5.6F) CAGAUACCCCAAGGAAUUUC | (9R) CAACGUGAAAUGAAGGAACG | Exons 5.6, 9 |

Primers 5.6F = Junction of exons 5 and 6.
